# Multidrug-resistant *Mycoplasma genitalium* infections in Europe

**DOI:** 10.1007/s10096-017-2969-9

**Published:** 2017-03-30

**Authors:** J. F. Braam, L. van Dommelen, C. J. M. Henquet, J. H. B. van de Bovenkamp, J. G. Kusters

**Affiliations:** 1 Department of Medical Microbiology, University Medical Center Utrecht, Heidelberglaan 100, 3584 CX Utrecht, The Netherlands; 2 PAMM Laboratory of Medical Microbiology, Veldhoven, The Netherlands; 3 Department of Dermatology, Catharina Hospital, Eindhoven, The Netherlands

## Abstract

In Japan and Australia, multidrug-resistant *Mycoplasma genitalium* infections are reported with increasing frequency. Although macrolide-resistant *M. genitalium* strains are common in Europe and North America, fluoroquinolone-resistant strains are still exceptional. However, an increase of multidrug-resistant *M. genitalium* in Europe and America is to be expected. The aim of this paper is to increase awareness on the rising number of multidrug-resistant *M. genitalium* strains. Here, one of the first cases of infection with a genetically proven multidrug-resistant *M. genitalium* strain in Europe is described. The patient was a native Dutch 47-year-old male patient with urethritis. *Mycoplasma genitalium* was detected, but treatment failed with azithromycin, doxycycline and moxifloxacin. A urogenital sample was used to determine the sequence of the 23S rRNA, *gyrA*, *gyrB* and *parC* genes. The sample contained an A2059G single nucleotide polymorphism (SNP) in the 23S rRNA gene and an SNP in the *parC* gene, resulting in an amino acid change of Ser83 → Ile, explaining both azithromycin and moxifloxacin treatment failure. The SNPs associated with resistance were probably generated de novo, as a link with high-prevalence areas was not established. It is, thus, predictable that there is going to be an increase of multidrug-resistant *M. genitalium* strains in Europe. As treatment options for multidrug-resistant *M. genitalium* are limited, the treatment of *M. genitalium* infections needs to be carefully considered in order to limit the rapid increase of resistance to macrolides and fluoroquinolones.

## Introduction

*Mycoplasma genitalium* infects between 1.0 and 3.5% of sexually active people in the general European population [1–3] and is slightly higher (up to 6.0%) in patients from sexually transmitted infection (STI) screenings [4–6]. Infection with *M. genitalium* causes non-gonococcal urethritis (NGU) in men [7] and urethritis, cervicitis [8], pelvic inflammatory disease (PID) [9, 10] and possibly tubal factor infertility in women [10, 11]. In Europe, azithromycin is currently the preferred therapy and, when this treatment fails, fluoroquinolones are used for second-line therapy [12].

Approximately 40% of the *M. genitalium* strains are resistant to azithromycin [13]. In addition, multidrug-resistant *M. genitalium* strains are frequently reported in the Pacific: in Australia, multidrug-resistant *M. genitalium* strains were identified in 9.8% [14] and in Japan in up to 30.8% of the patients screening for STIs [15]. While in the United States and Europe macrolide-resistant strains are common, fluoroquinolone-resistant strains are still quite exceptional. The first genetically proven fluoroquinolone-resistant strain in Europe was described by Pond et al. in the UK [16]. This strain possessed no macrolide resistance-associated mutation, since it could be treated successfully with azithromycin and the patient had no symptoms during the 5-month follow-up period. It is to be expected that an increasing number of the circulating *M. genitalium* strains in Europe would be resistant to both macrolides and fluoroquinolones. However, there are almost no peer-reviewed reports on the occurrence of multidrug resistance in Europe or the United States of America. To our knowledge, only a single European case of infection with a multidrug-resistant strain has been reported and, unfortunately, the genetic basis of this multidrug-resistant isolate was not described [17].

Resistance to macrolides is associated with mutations at positions 2058 and 2059 (*Escherichia coli* numbering) in region 5 (V-region) of the 23S rRNA gene [18]. Moxifloxacin resistance has been described to result from mutations in the *gyrA* and *parC* genes [16]. In this article, one of the first genetically documented European cases of a multidrug-resistant *M. genitalium* infection that has resulted in treatment failure is described.

## Materials and methods

*Mycoplasma genitalium* was detected in a native Dutch 47-year-old male patient with urethritis. The patient repeatedly tested negative by routine polymerase chain reaction (PCR) assays for *Chlamydia trachomatis*, *Neisseria gonorrhoeae* and *Trichomonas vaginalis*, and no other pathogen was detected using culture. The patient was initially treated with a single dose of 1 g of azithromycin. When this failed, he consecutively received azithromycin for 5 days, moxifloxacin 400 mg once daily for 10 days, doxycycline 100 mg twice daily for 7 days and, ultimately, moxifloxacin again 400 mg once daily for 14 days. After finishing the last antibiotic treatment, the patient still complained of an intermittent burning sensation and minimal penile discharge. The patient did not have sexual intercourse during the last months of treatment. Since azithromycin and moxifloxacin resistance was suspected, the latest urogenital sample obtained four weeks after completion of this cure was used for determining the sequence of the 23S rRNA gene according to the method of Nijhuis et al. [19] and *gyrA*, *gyrB* and *parC* genes according to Pond et al. [16].

## Results and discussion

The strain harboured an A2059G mutation in the 23S rRNA gene. This mutation is known to result in azithromycin resistance [18] and, thus, explains the azithromycin resistance of this strain. In addition, a single nucleotide polymorphism (SNP) in the *parC* gene resulting in an amino acid change of Ser83 → Ile was observed, explaining the resistance to moxifloxacin [16]. We cannot completely exclude that the obtained SNPs result from a mixed infection with one strain that is resistant to azithromycin but sensitive to moxifloxacin, and a second strain that is sensitive to azithromycin and resistant to moxifloxacin. This is unlikely, as we sequenced directly on the PCR products obtained from the urogenital sample and only a single peak was detected at the positions of these two resistance-associated SNPs. In addition, the infection could not be resolved by antibiotic treatment and sequences were obtained from a sample after treatment of the patient with azithromycin and moxifloxacin, making it very likely that both SNPs exist in the same isolate. Both resistance mechanisms are based on single-point mutations and, as the patient had no known contacts with individuals of Asian or Pacific origin, it is, therefore, most likely that these SNPs were derived de novo.

Resistance to macrolides in *M. genitalium* is rapidly increasing. Most countries use a single-dose macrolide for the treatment of both *C. trachomatis* and *M. genitalium*. While still effective for the treatment of *C. trachomatis*, the efficacy of 1 g of azithromycin for *M. genitalium* has decreased from 85.3% prior to 2009 to 67.0% after 2009, and is now as low as 60.0% [13]. This rapid increase in resistance to macrolides can partly be explained by the resistance mechanism of *M. genitalium*, where a change of only one single nucleotide results in resistance to azithromycin and related macrolide drugs.

Although a bit more complex, an SNP in the *gyrA*, *gyrB* and *parC* genes can result in fluoroquinolone resistance [20]. In Japan, fluoroquinolones (in particular, levofloxacin) are frequently used for the treatment of *C. trachomatis* [21]. As levofloxacin is not very effective against *M. genitalium* [22, 23], treatment results in induction of quinolone resistance in *M. genitalium* strains potentially present in these patients. As a consequence, fluoroquinolone resistance in Japan is rapidly increasing. One study revealed that the prevalence of fluoroquinolone resistance with proven SNP in the *parC* gene in Japan between 2006 and 2008 was 10.7% [24]. This prevalence further increased to 47.1% in 2013 [21]. In other areas in the Pacific, a similar increase in fluoroquinolone resistance has been reported [25]. As the first-choice regimens for treating *M. genitalium* are based on either azithromycin or moxifloxacin (or closely related macrolides/fluoroquinolones) and alternative treatment options are limited, isolates resistant to azithromycin and moxifloxacin will be very difficult to eradicate [12]. Such multidrug-resistant *M. genitalium* strains can effectively be treated with pristinamycin [26]. However, pristinamycin is expensive and not widely available [25], which makes the treatment of multidrug-resistant *M. genitalium* challenging.

To the best of our knowledge, we describe here one of the first genetically documented multidrug-resistant *M. genitalium* strains in Europe. Since microbiological cure was not expected with any other available antibiotic in our patient and the patient eventually had only minor complaints, he declined further treatment. The origin of the infecting strain seemed to be in The Netherlands and was probably derived from de novo mutations selected by antibiotic therapy. Since macrolide resistance has increased over time in *M. genitalium*, it seems plausible that fluoroquinolone resistance will become more prevalent in the near future. The efficacy of current treatment protocols for *M. genitalium* is, therefore, seriously threatened, since treatment options are limited. Guideline-based treatment and restrictive antibiotic use are crucial in limiting antibiotic resistance. Future studies are needed to assess the clinical impact of emerging fluoroquinolone resistance in Europe.
